# Modelling the co-evolution of indirect genetic effects and inherited variability

**DOI:** 10.1038/s41437-018-0068-z

**Published:** 2018-03-28

**Authors:** Jovana Marjanovic, Han A Mulder, Lars Rönnegård, Piter Bijma

**Affiliations:** 1 Wageningen University and Research, Animal Breeding and Genomics, PO Box 338, 6700 AH Wageningen, The Netherlands; 2 Department of Animal Breeding and Genetics, Swedish University of Agricultural Sciences, Box 7023, 75007 Uppsala, Sweden; 3 Dalarna University, School of Technology and Business Studies, 79188 Falun, Sweden

## Abstract

When individuals interact, their phenotypes may be affected not only by their own genes but also by genes in their social partners. This phenomenon is known as Indirect Genetic Effects (IGEs). In aquaculture species and some plants, however, competition not only affects trait levels of individuals, but also inflates variability of trait values among individuals. In the field of quantitative genetics, the variability of trait values has been studied as a quantitative trait in itself, and is often referred to as inherited variability. Such studies, however, consider only the genetic effect of the focal individual on trait variability and do not make a connection to competition. Although the observed phenotypic relationship between competition and variability suggests an underlying genetic relationship, the current quantitative genetic models of IGE and inherited variability do not allow for such a relationship. The lack of quantitative genetic models that connect IGEs to inherited variability limits our understanding of the potential of variability to respond to selection, both in nature and agriculture. Models of trait levels, for example, show that IGEs may considerably change heritable variation in trait values. Currently, we lack the tools to investigate whether this result extends to variability of trait values. Here we present a model that integrates IGEs and inherited variability. In this model, the target phenotype, say growth rate, is a function of the genetic and environmental effects of the focal individual and of the difference in trait value between the social partner and the focal individual, multiplied by a regression coefficient. The regression coefficient is a genetic trait, which is a measure of cooperation; a negative value indicates competition, a positive value cooperation, and an increasing value due to selection indicates the evolution of cooperation. In contrast to the existing quantitative genetic models, our model allows for co-evolution of IGEs and variability, as the regression coefficient can respond to selection. Our simulations show that the model results in increased variability of body weight with increasing competition. When competition decreases, i.e., cooperation evolves, variability becomes significantly smaller. Hence, our model facilitates quantitative genetic studies on the relationship between IGEs and inherited variability. Moreover, our findings suggest that we may have been overlooking an entire level of genetic variation in variability, the one due to IGEs.

## Introduction

Social interactions are common in nature, and other individuals are usually the most important part of the environment experienced by an individual (Wolf 2003; Frank 2007). The environment created by social partners through actions, such as competition or cooperation, is referred to as the social environment. Variation in the quality of the social environment may originate partly from genetic variation in the social partners, which would make the social environment heritable (Wolf et al. 1998). The classical example of a heritable environment is the one provided by a mother to her offspring in mammals (Dickerson 1947; Willham 1963; Falconer 1965; Kirkpatrick and Lande 1989; Cheverud 2003).

In the traditional quantitative genetic model, the phenotype of an individual is the sum of the direct effect of its own genes (DGE) and an environmental effect. However, because the environmental effect includes a component due to the social environment, the phenotype of an individual is also a function of the genes of its social partners. The heritable effect of a social partner on the trait value of the focal individual is known as an Indirect Genetic Effect (IGE) (Griffing 1967). IGEs have consequences for trait values and fitness of individuals that interact, and subsequently for the course of the evolutionary processes (e.g., Hamilton 1964a; Moore et al. 1997; Wolf et al. 1998).

In the field of animal breeding, interest in social interactions has increased in recent decades, as both theoretical and empirical studies show that not only fitness but also trait values of individuals can be affected by genes of other individuals (Muir 2005; Bijma et al. 2007a, b). IGEs have been studied in both animal and plant populations, and in a number of those studies social interactions contributed substantially to heritable variation in the trait (reviewed by Ellen et al. 2014). Well-known cases of IGEs in domestic animals include cannibalistic behavior in laying hens, which causes mortality (Muir 1996; Ellen et al. 2008), competition and tail biting in pigs, which is associated with poorer growth (Arango et al. 2005; Camerlink et al. 2013, 2014; Bergsma et al. 2013), and aggression and competition in fish species such as Nile tilapia and Atlantic cod, which reduces growth (Nielsen et al. 2014; Khaw et al. 2016).

In addition to the effects of social interactions on trait values, it has been observed in aquaculture populations that competition for feed and formation of social hierarchies also inflates trait variability (Jobling 1995; Cutts et al. 1998; Hart and Salvanes 2000). Because this pattern is so evident, variability in body weight among individuals has become a standard measure of the degree of competition in aquaculture; the degree of competition is measured by the coefficient of variation (CV) of body weight, where a high CV indicates strong inter-individual competition (Jobling 1995). In farmed fish populations, the CV is usually between 20 and 60 % (Gjedrem 2000; Ponzoni et al. 2005; Gjedrem and Baranski 2009), which suggests moderate to strong competition.

Indications of a close relationship between competition and variability are also coming from the field of plant breeding, where breeders have successfully improved productivity of crops by selecting, partly unintentionally, less competitive phenotypes, which also resulted in more uniform crops (Donald 1968; Austin et al. 1980; Denison et al. 2003). Moreover, the connection between yield, competition, and variability has also been made in game theory, where it was shown that the lowest competition and highest yield is achieved when plants are phenotypically uniform (Zhang et al. 1999). Hence, in plants, there is clear evidence of a genetic relationship, where reduced competition leads to less variability and higher yield.

The variability of trait values of a genotype, measured either repeatedly on the same individual, or on multiple individuals belonging to the same family, has been studied as a quantitative trait in its own right. This trait is often referred to as “inherited variability” or “heritable variation in environmental variance” (SanCristobal-Gaudy et al. 1998; Mulder et al. 2008; Hill and Mulder 2010). The study of variability has been a part of quantitative genetics for several decades already, but it has gained particular attention in recent years due to the development of new methods to estimate genetic variance in variability (SanCristobal-Gaudy et al. 1998; Sorensen and Waagepetersen 2003; Mulder et al. 2009; Rönnegård et al. 2010) and substantial empirical evidence for a genetic basis of variability in livestock, aquaculture, and laboratory populations (reviewed by Hill and Mulder 2010). In several fish populations, for example, it has been found that variability of body weight has a large genetic component (Janhunen et al. 2012; Sonesson et al. 2013; Khaw et al. 2015; Sae-Lim et al. 2015a, b; Marjanovic et al. 2016). However, despite the clear relationship between competition and variability observed at the phenotypic level, inherited variability has not been connected to competition in quantitative genetic models.

As social interactions are often a source of IGEs, the observed relationship between competition and variability on the phenotypic level (Jobling 1995; Cutts et al. 1998; Hart and Salvanes 2000; Denison et al. 2003) strongly suggests an underlying genetic relationship between the two phenomena. At present, little is known of this genetic relationship, both in plants and animals, which may be due to a lack of quantitative genetic models that connect both phenomena. On the one hand, current quantitative genetic models of inherited variability ignore social interactions, since they treat variability as a trait of the focal individual only, ignoring the contribution of social partners. On the other hand, standard IGE-models cannot explain the relationship between competition and variability, since phenotypic variance is independent of the level of IGEs in those models. However, by ignoring IGEs, we may be overlooking an important component of heritable variation in trait variability.

The joint study of IGEs and inherited variability could help us understand observations from animal and plant breeding, and possibly enable utilization of genetic variation that has so far been untapped. In addition, it may bring new insight in mechanisms of canalization or insensitivity of individuals to genetic and environmental changes (Waddington 1942), and broaden our understanding of phenotypic evolution. Therefore, a joint study of IGEs and variability could make a significant contribution to the field of quantitative genetics, and its applications in animal and plant breeding and in evolutionary biology.

As a first step towards unraveling the genetic relationship between social interactions and inherited variability, we present a quantitative genetic model that integrates both phenomena. We use Monte Carlo simulation to evaluate the behavior of the model, and demonstrate that the model mimics the co-evolution of social interactions and variability observed in phenotypic studies.

## Theory

### Model

The genetics of socially affected traits can be studied using two approaches; variance component models or trait-based models (McGlothlin and Brodie 2009; Bijma 2014). In variance component models, the individual phenotype is divided into a direct genetic component originating from the focal individual, and an indirect genetic component originating from its social partner (Griffing 1967). In this approach, it is not needed to know which traits are causing the IGE. Instead, DGEs and IGEs are estimated as random effects using linear mixed models and information on genetic relationships between individuals (Muir 2005; Bijma et al. 2007b). See Table 1 for notation.

**Table 1:** Notation key

Symbol/abbreviation	Meaning
DGE, IGE	Direct genetic effect, indirect genetic effect
*i, j*	Focal individual, group mate of individual *i*
*P*_*t*, *GR*_	Body weight in the current time point
*P*_*t*−1 *,GR*_	Body weight in the previous time point
*μ*_*GR*_	Mean growth rate
*A*_*GR*_	Breeding value for growth rate
*A*_*D*_	Direct breeding value for *b*—genetic resistance to competition
*A*_*I*_	Indirect breeding value for *b*—genetic cooperation effect
*E*_*p,GR*_, *E*_*t,GR*_	Permanent and temporary environmental effects on growth rate
*E*_*D*_ *, E*_*I*_	Direct and indirect environmental effects for *b*
*b*	Regression coefficient
$\overline b$	Average regression coefficient
*b* value	Regression coefficient that affects the phenotype of the focal individual
${{\sigma }}_{{{A}}_{{{GR}}}}^2$, ${\sigma }_{{A}_{{D}}}^2$, ${\sigma }_{{A}_{{I}}}^2$	Genetic variance for growth rate, direct and indirect genetic variance for *b*
${\sigma }_{{E}_{{{p}},{{GR}}}}^2$, ${\sigma }_{{E}_{{{t}},{{GR}}}}^2$	Permanent and temporary environmental variance for growth rate
${\sigma }_{{E}_{{D}}}^2$, ${\sigma }_{{E}_{{I}}}^2$	Direct and indirect environmental variance for *b*
${\sigma} _{P_{{{GR}}}}^2$	Phenotypic variance of growth rate
*h* ^2^	Heritability of growth rate

The trait-based models, in contrast, define IGEs on the phenotype of the focal individual as a function of trait values of its social partners (Moore et al. 1997; Wolf et al. 1998; Bijma 2014). In this case, the traits causing the indirect effects need to be identified. When interaction is between two individuals, and the target trait and the trait causing the IGE, also known as the “effector trait”, are the same, the trait-based model can be written as (Moore et al. 1997)

1 $$P_i = A_i + e_i + \psi P_j,$$

where *P*_*i*_ is the phenotypic value of the focal individual *i*, *A*_*i*_ is the additive genetic effect originating from the focal individual, *P*_*j*_ is the phenotypic value of its social partner *j*, *ψ* is the “regression coefficient” of *P*_*i*_ on *P*_*j*_, and *e*_*i*_ is a residual. (With feedback, i.e., when trait levels of interacting individuals are reciprocally affected, *ψ* is not a true regression coefficient; see Bijma 2014). We will use this model and observations from aquaculture as a starting point to draw a connection between IGEs and inherited variability.

Phenotypic studies in aquaculture suggest that the behavior of a fish towards its social partners depends on its size relative to that of its partners, where larger fish are usually dominant and aggressive, while smaller fish are subordinate and submissive (Doyle and Talbot 1986; Huntingford et al. 2012). In anemonefish, for example, large individuals are dominant members of social groups and display aggressive behavior towards subordinates (Fricke and Fricke 1977; Iwata et al. 2008). Similarly, Oscars (cichlid fish, *Astronotus ocellatus*) chase and attack smaller conspecifics, but avoid larger individuals (Beeching 2010). Difference in body weight, therefore, affects phenotypes of the interacting individuals, with higher body weight giving a competitive advantage to the individual in terms of growth rate. Thus, to account for the competitive effect of body weight on growth rate, we need to model the evolution of body weight over the life of the interacting individuals.

Therefore, we developed a basic quantitative genetic model involving interactions of two individuals. In this model, our target trait is growth rate between time point *t*−1 and *t*, while the effector trait is the difference in body weight between the individuals that interact at the previous time point *t−1*. The change in body weight, i.e., growth rate, of the focal individual is a function of genetic and environmental effects of the focal individual itself on its growth rate, and of the difference in body weight between the social partner and the focal individual, multiplied by a regression coefficient,

2 $$\begin{array}{l}P_{t,i} - P_{t - 1,i} = \\ \mu _{{{GR}}} + A_{{{GR}},i} + E_{p,{{GR}},i} + E_{t,{{GR}},i} + b_{ij}\left( {P_{t - 1,j} - P_{t - 1,i}} \right)\end{array}$$

where *P*_*t*,*i*_ is the body weight of focal individual *i* at time point *t*, *P*_*t*−1,*i*_ is body weight of *i* at the previous time point, *μ*_*GR*_ is the mean growth rate of the population, *A*_*GR*,*i*_ is a (direct) breeding value for growth rate of individual *i*, *E*_*p*,*GR*,*i*_ and *E*_*t*,*GR*,*i*_ are permanent and temporary non-heritable (“environmental”) effects of individual *i*, and *b*_*ij*_ is a regression coefficient.

### The meaning of *b*_*ij*_

The *b*_*ij*_ in our model measures the effect of a difference in body weight between the social partner and the focal individual on the growth rate of the focal individual. Hence, the absolute value of *b*_*ij*_ reflects the strength of the social interaction. When *b*_*ij*_ is negative, growth rate of individual *i* is reduced when *j* has higher body weight than *i*, indicating competition. Conversely, when *b*_*ij*_ is positive, growth rate of *i* is increased when *j* has higher body weight than *i*, indicating cooperation, i.e., “helping the one who lags behind” (Box 1). Thus, *b* is a measure of cooperation; negative *b* indicates competition, positive *b* cooperation, and an increase in *b* an increase of cooperation (i.e., less competition). The model described by Eq. 2 can be written in matrix form for both individuals simultaneously, which may facilitate analysis of the behavior of the model (Appendix A).

### Genetic variation in *b*

Trait-based IGE models usually assume that the “regression coefficient” *ψ* is constant within a population (Eq. 1). However, several empirical studies that were able to estimate *ψ*, show that it may differ between genotypes (Kent et al. 2008; Bleakley and Brodie 2009; Chenoweth et al. 2010). Hence, empirical studies suggest that *ψ* shows genetic variation, and can thus respond to selection. Following this evidence, we allow *b* to evolve. Therefore, *b* is not a fixed parameter, but specific for every interacting couple. We propose that heritable variation in *b* is a result of a direct genetic effect of the focal individual (*A*_*D*,*i*_), representing resistance to competition, and an indirect genetic effect of its social partner, representing cooperative effect (*A*_*I*,*j*_). While *b* is a property of both the focal individual and its social partner, it affects the phenotype of the focal individual only; we will therefore refer to this *b* as “the *b* value of the focal individual”. Thus, for focal individual *i* with social partner *j*, the regression coefficient *b*_*ij*_, i.e., the *b* value of the focal individual, is given by

3 $$b_{ij} = \bar b + A_{{{D}},i} + E_{{{D}},i} + A_{{{I}},j} + E_{{{I}},j},$$

where $\bar b$ represents the average regression coefficient, which is a population parameter that is negative under competition and positive under cooperation. The *A*_*D*,*i*_ and *E*_*D*,*i*_ are the direct genetic and environmental effect of individual *i* on *b*_*ij*_, while *A*_*I*,*j*_ and *E*_*I*,*j*_ are the indirect genetic and environmental effect of individual *j* on *b*_*ij*_. Appendix B contains extension of Eq. 2 to accommodate larger group size.

### Inherited variability

Note that our model does not include an explicit breeding value for inherited variability. Instead, as shown in the section “Simulation” below, genetic variation in variability is an emerging property of the model, resulting from genetic effects of competition, i.e., the direct and indirect breeding values for *b*. In other words, our model shows that heritable effects on competition result in inherited variability. In the “Discussion” section, we further investigate how breeding values for *b* correlate with direct and indirect breeding values for inherited variability (see section “Estimating *b*” below; see also section “Breeding values for *b* and variability”).

### Competition, cooperation, and the sign of ${\bar{\boldsymbol b}}$

**Fig. 1:**
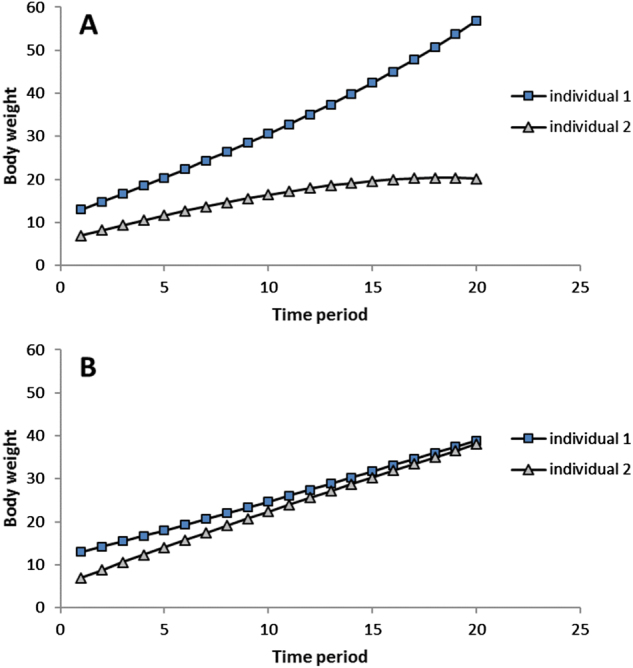
Expected growth curves of two individuals in a group under competition (**a**) and cooperation (**b**)

We use the term “competition” to describe the situations where the larger individual continues to increase in size, while the smaller individual lags behind, leading to divergence of their body weights through time (Fig. 1a). This is typical for populations where $\bar b$ < 0. We use the term “cooperation” to describe the situation where individuals become increasingly similar in body weight over time (Fig. 1b). This occurs when growth rate of the larger individual decreases, while the smaller one catches up. This is typical for populations, where $\bar b$ > 0.

### Asymmetry: *b*_*ij*_ vs. *b*_*ji*_

Note that we distinguish between resistance to competition (*A*_*D*_) and cooperativeness (*A*_*I*_), as these may be different properties of an individual. For example, consider the pair *i* and *j* in a population showing competition ($\bar b$ < 0). Suppose that *i* is very competitive (*A*_*I*,*i*_ < 0) and also resistant to competition (*A*_*D*,*i*_ > 0), while *j* is very cooperative (*A*_*I*,*j*_ > 0) but very sensitive to competition (*A*_*D*,*j*_ < 0). Then the effect of *j* on *i* will be small, while the effect of *i* on *j* will be large (Supplementary File 1, grey cells in Table S2). In other words, an individual that is strongly affected by its social partner does not necessarily also have a strong effect on its social partner. Hence, *b* is non-symmetric, i.e., *b*_*ij*_ ≠ *b*_*ji*_.

### Simulation

We used Monte Carlo simulation to investigate whether our model (Eq. 2) predicts the empirically observed relationship between competition and variability, and whether methods for selection against competition (e.g. group selection) also result in a reduction of variability. We considered five values for the average value of *b*$(\bar b)$, to which we refer as scenarios (Table 2).

**Table 2:** Parameters used in simulation

Parameters	Scenarios				
	Competition		Neutral	Cooperation	
	1	2	3	4	5
Mean growth rate, *μ*_G*R*_			10 g		
Genetic variance for growth rate, ${\sigma }_{{{A}}_{{{{GR}}}}}^2$			1 g^2^		
Permanent environmental variance, ${\sigma }_{{E}_{{{p}},{{{GR}}}}}^2$			0.4 g^2^		
Temporary environmental variance, ${\mathrm{\sigma }}_{{{E}}_{{{t}},{{GR}}}}^2$			0.6 g^2^		
Cooperation effect, $\bar b$	−0.08	−0.05	0	0.05	0.08
Direct and indirect genetic and environmental variance, ${\sigma }_{{A}_{{D}}}^2 = \sigma _{A_{{I}}}^2 = \sigma _{E_{{D}}}^2 = \sigma _{E_{{I}}}^2$			0.225 × 10^−3^		
Phenotypic variance, $\sigma _{P_{{{GR}}}}^2$			2 g^2^		

*$\sigma _{P_{{{GR}}}}^2$ is calculated excluding *b*, i.e., as $\sigma _{P_{{{GR}}}}^2 = {{\sigma }}_{{{A}}_{{{GR}}}}^2 + {{\sigma }}_{{{E}}_{{{p}},{{GR}}}}^2 + {{\sigma }}_{{{E}}_{{{t}},{{GR}}}}^2$

**The scenarios only differ in the input values for the cooperation effect, while other values are equal for all scenarios

Negative values of $\bar b$ correspond to competition (Scenario 1-strong competition; Scenario 2-moderate competition), while positive values reflect cooperation (Scenario 4-moderate cooperation; Scenario 5-strong cooperation). Scenario 3 represents a neutral environment with $\bar b$ = 0.

The genetic values of all individuals in the population were simulated as inherited from their parents (base population), assuming Mendelian inheritance, while their environmental values were sampled from independent normal distributions. All individuals were randomly assigned to groups of 2 members. Phenotypes were constructed for 10 time points using Eqs. 2 and 3. Average starting weight was 10 g, and average growth rate between time points was also 10 g. Hence, to illustrate the behavior of our model as simple as possible, we considered absolute growth. Obviously, for the analysis of real data, a more biologically realistic growth model, such as relative growth, may be used. With relative growth, the impact of competition may be higher, since individuals may diverge more in scenarios with competition, as larger individuals grow faster, while smaller individuals slower, compared to absolute growth (see Supplementary file 2 for two small simulation examples).

For each scenario, there were 100 replicates. Table 2 contains parameter values used in the simulation. Appendix C contains a detailed description of the simulation procedure.

### Relationship between ${\bar{\boldsymbol b}}$ and variability

**Fig. 2:**
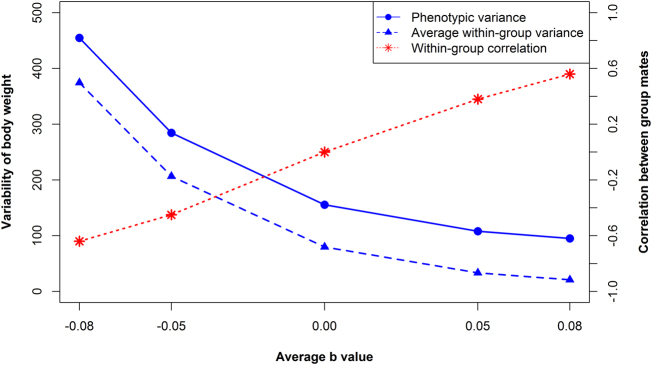
Variability of body weight in a population and correlation between group mates across five scenarios i.e., five average *b* values ($\overline b$). Variability is expressed as the average within-group variance of body weight of two group mates and as overall phenotypic variance in the whole population

The relationship between competition and variability generated by our model was assessed at two levels. First, we considered the average within-group variance of body weight at the last time point. Second, we considered the overall phenotypic variance in the entire population. Results are presented in Fig. 2 as averages over 100 replicates.

Across the five scenarios, both average within-group variance and phenotypic variance decreased curvilinear with increasing $\bar b$, i.e., with increasing cooperation (Fig. 2). The average within-group variance ranged from 376.4 g^2^ (sd, ±14.4 g^2^) to 20.9 g^2^ (sd, ±0.7 g^2^), which is an 18-fold difference in variability of body weight between scenarios 1 and 5. The phenotypic variance ranged from 457.3 g^2^ (sd, ±15.7 g^2^) to 95.1 g^2^ (sd, ±2.6 g^2^), showing a 5-fold difference in variability between scenarios 1 and 5. These results show that our model results in a relationship between competition ($\overline b$) and variability that is also found in real data.

The difference between the average within-group variance and the phenotypic variance is related to the similarity of group mates. Total phenotypic variance is the sum of between- and within-group variance. When group mates are independent and group size equals two, the average within-group variance is half of the phenotypic variance. Average within-group variance, however, was much larger than half of the phenotypic variance in scenarios with negative *b*, but much smaller in scenarios with positive *b*. The correlation between group mates is calculated as $\rho = \frac{{\sigma _b^2 - \sigma _w^2}}{{\sigma _b^2 + \sigma _w^2}}$, where $\sigma _b^2$ is between group variance and $\sigma _w^2$ is within-group variance. In scenarios with negative *b*, the correlation between group mates was negative, which means that group mates were dissimilar in the competitive environment (Fig. 2). When *b* was positive, correlation between group mates was positive, indicating higher similarity of group mates in the cooperative environment (Fig. 2). For $\bar b$ = 0, the average within-group variance was approximately one half of the phenotypic variance.

### Growth curve patterns in relation to *b* values

In this section, we look into how variation in *b* around its average, affects the variability among group mates. Within every scenario (Table 2) $\bar b$ was the same for all individuals; however, variation in *b* values of individuals existed due to variation in direct and indirect genetic and environmental components that make up *b* (Eq. 3). Therefore, in every scenario some groups would have individuals that both have high *b* values, some groups would have individuals with low *b* values, and variations in between. We hypothesize that group mates that both have high *b* values, i.e., that are both cooperative and resistant to competition, grow more uniform compared to those with low *b* values, i.e., group mates that are both competitive and sensitive to competition.

**Fig. 3:**
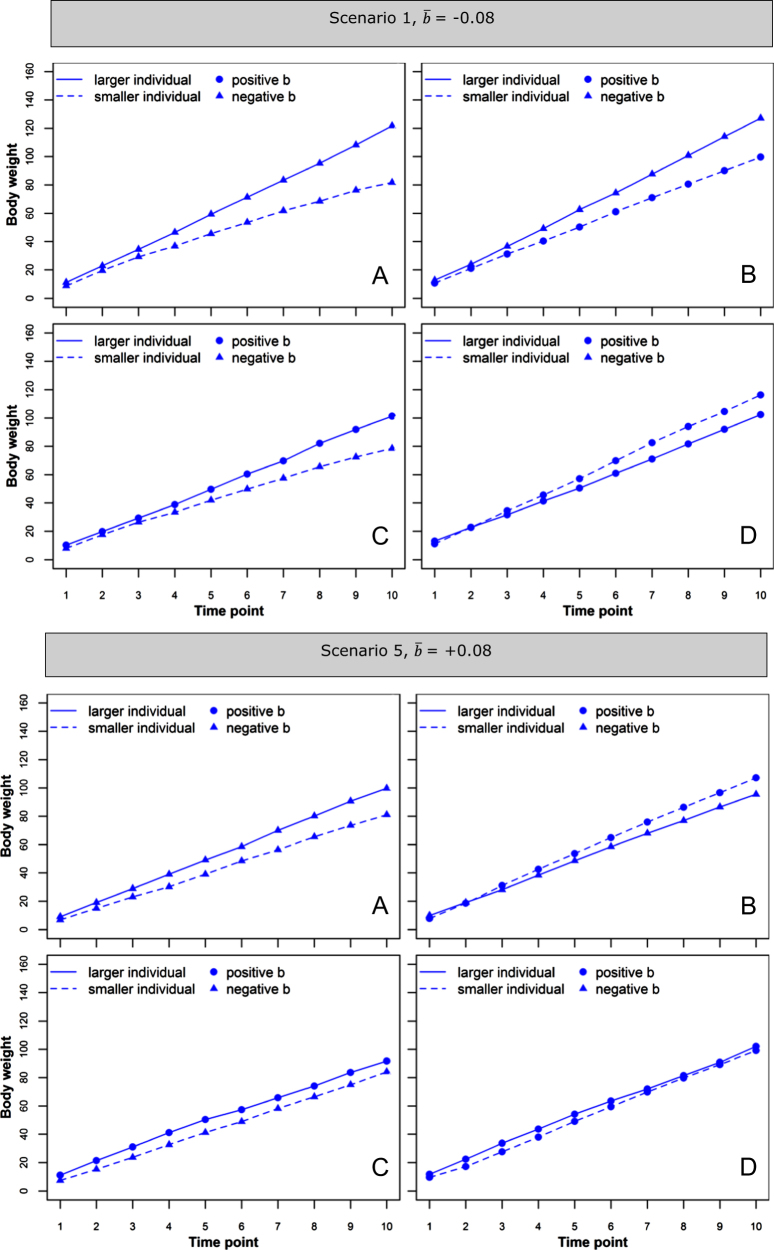
Growth curves of two group mates (one larger than the other) that have lowest sum of *b*’s (**a**); the initially larger individual has negative *b*, the smaller one has positive *b* (**b**); the initially larger individual has positive *b*, the smaller one has negative *b* (**c**); lowest sum of *b*’s (**d**), for scenarios 1 and 5. Each panel shows one typical replicate

To illustrate this, we selected groups that have individuals with the highest and the lowest *b* values for each of the scenarios. An additional condition when selecting groups was that individuals have an initial difference in their body weight of ~2 sd. The growth curves in relation to the level of *b* values within a group are illustrated in Fig. 3a, d for scenario 1 ($\bar b$ = −0.08, strong competition) and 5 ($\bar b$ = +0.08, strong cooperation). Results for scenarios 2–4 are presented in Supplementary File 3. Supplementary file 4 contains *b* values of individuals from all the scenarios.

In both scenarios 1 and 5, individuals in a group with the low *b* values differed substantially in their final body weight (Fig. 3a). Individuals with the high *b* values, however, maintained a similar body weight through time (Fig. 3d), which is in agreement with our hypothesis.

We also looked into groups that had individuals with positive/negative combinations of *b* values. When the initially larger individual had a negative *b* value, its body weight increased over time, resulting in a larger size difference between the two group mates, unless the smaller individual had a positive *b* value, which allowed it to catch up (Fig. 3b). Similarly, the size difference decreased when the larger individual had a positive *b* value, even when the smaller individual had a negative *b* value (Fig. 3c). It was also possible to get re-ranking of the individuals, i.e., the smaller individual can become the larger one. This can happen for example when the smaller individual has a high positive *b* value, while the larger individual has a low negative *b* value (Scenario 5, Fig. 3b).

Expressions (Appendix A) for the expectation of the difference in the phenotypic values and the variance of this difference at time point *T*, i.e. *E*(*P*_*T*__*,*__*i*_ − *P*_*T*__*,*__*j*_|*b*_*ij*_,*b*_*ji*_) and *V*(*P*_*T*,*i*_ − *P*_*T*__*,*__*j*_|*b*_*ij*_,*b*_*ji*_), demonstrate that the phenotypic variance within a group is directly related to the sum of *b* values within the group. The expressions show that the expected difference is zero if there is no initial difference at *T* = 0, while the variance depends directly on the sum of *b*_*ij*_ and *b*_*ji*_. More details can be found in Appendix A.

### Breeding values for *b* and variability

**Fig. 4:**
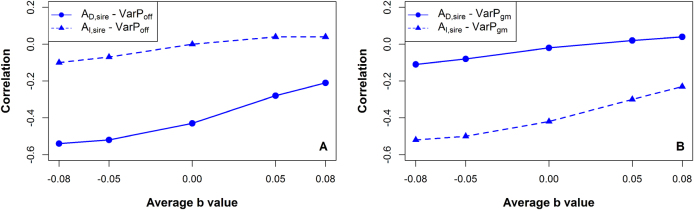
**a** Correlation (r) between direct (*A*_*D*_) and indirect (*A*_*I*_) breeding value of a sire and variance of body weight of his offspring (*VarP*_*off*_) for each of the five scenarios, i.e., average *b* values ($\overline b$). **b** Correlation (r) between direct (*A*_*D*_) and indirect (*A*_*I*_) breeding value of a sire and variance of body weight of the group mates of its offspring (*VarP*_*gm*_), for each of the five scenarios, i.e., average *b* values $\left( {\overline b } \right)$

If a connection between competition and variability exists not only on the phenotypic level but also on genetic level, we should see less variation in body size among the offspring of sires that have positive direct breeding values (*A*_*D*_) for *b*, as these individuals should be more resistant to competition. This links our model to the definition of inherited variability, where parents with low breeding values for variability have offspring with lower phenotypic variance. Figure 4a indeed shows that the correlation between *A*_*D*_ of sires for *b* and variability of body weight of their offspring is negative, ranging from −0.55 (sd, ±0.07) to −0.20 (sd, ±0.09) across scenarios. This suggests that individuals that are genetically more resistant to competition are less variable. Moreover, offspring of sires with positive indirect breeding values (*A*_*I*_) for *b* should be less competitive. The group mates of these “social” individuals should therefore show less variability compared to group mates of individuals with negative indirect breeding values for *b*. In other words, *A*_*I*_ of a sire affects the variability of phenotypes of the group mates of his offspring. As expected, Fig. 4b shows negative correlations between *A*_*I*_ of sires and variability of the group mates of their offspring. Figure 4a,b also shows a small negative correlation between *A*_I_ of a sire and variability of his offspring, and between *A*_*D*_ of a sire and variability of the group mates of its offspring. This result suggests a second-order effect; for the direct effect, for example, the *A*_*D*_ of a sire first affects the trait values of its own offspring, which subsequently affects the variability of their groups mates in the next time period. For standard errors of the correlations see Supplementary file 5.

### Selection

Individual selection has often been used with great success for improvement of livestock and aquaculture traits. However, this type of selection ignores the contribution of IGE which may hamper the improvement of socially affected traits. An alternative strategy is a group selection, which takes indirect genetic effects into account (Griffing 1976).

**Fig. 5:**
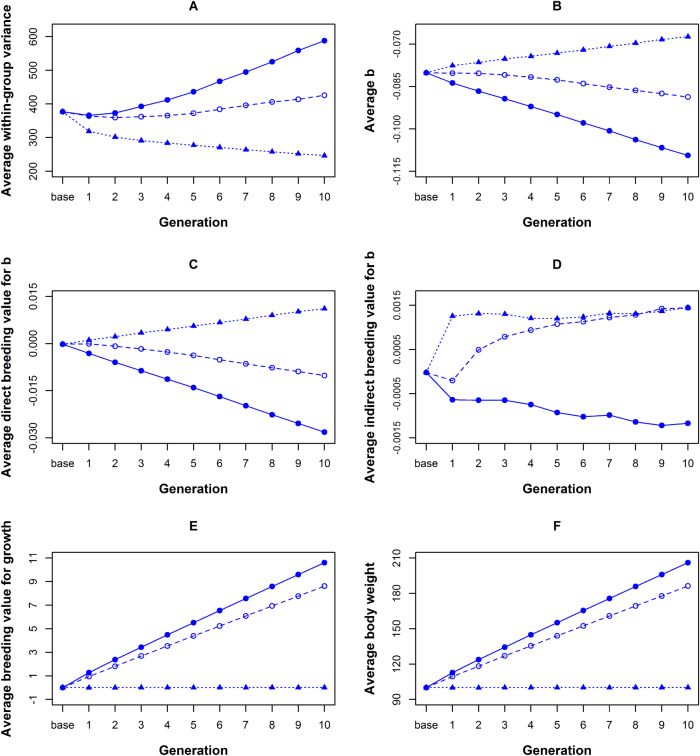
Effect of three types of selection on average within-group variance (**a**), average *b* ($\overline b$) (**b**), average direct breeding value for *b* (**c**), average indirect breeding value for *b* (**d**), average breeding value for growth (**e**), and average body weight (**f**), in the population

To see how variability responds to selection, and whether we can capture direct (*A*_*D*_) and indirect genetic effects (*A*_*I*_) for *b* with existing selection methods, we performed three types of selection: individual selection for body weight, group selection for body weight, and group selection for lower variance of body weight. In all three cases, selection was done using observations from time point 10. With individual selection, the 11 % of the heaviest individuals were selected as parents of the next generation. With group selection for body weight, the individuals from the 11 % of groups with the highest average body weight were selected. With group selection for lower variance, the individuals from the 11 % of groups with the lowest variance in body weight were selected. We illustrate the effect of selection by using base population with $\bar b$ = −0.08 (Scenario 1—strong competition, Table 2). Selections were performed for 10 generations. Correlations between *A*_*D*_ and *A*_*I*_, *A*_*D*_ and *A*_*GR*_, and *A*_*I*_ and *A*_*GR*_, were all set to 0. See Appendix C for further details. Figure 5 presents the results as averages over 100 replicates. For standard errors see Supplementary file 6.

Individual selection increased mean body weight (Fig. 5f), but also decreased *A*_*D*_ (Fig. 5c) and *A*_*I*_ (Fig. 5d), causing an increase in variability in the population (Fig. 5a). In other words, individual selection increased variability.

Both types of group selection increased *A*_*I*_ (Fig. 5d), suggesting that group selection at least partially exploited genetic differences in indirect genetic effects on *b*. Variability of body weight decreased when group selection was made on variance, but increased slightly when group selection was for average body weight, however much less compared to individual selection (Fig. 5a). This increase in variability with group selection for average body weight originated from a decrease in *A*_*D*_. With group selection on variance, in contrast, *A*_*D*_ increased (Fig. 5c). Group selection on the variance, therefore, captured direct and indirect genetic effects on *b* better than group selection on the average body weight. Group selection on the variance did not change mean body weight, because the correlations between *A*_*D*_ and *A*_*GR*_, and *A*_*I*_ and *A*_*GR*_ were zero (Fig. 5f). Group selection for average body weight, on the other hand, increased mean body weight in magnitude similar to individual selection (Fig. 5f).

## Discussion

We have proposed a quantitative genetic model that integrates competition and variability, and have shown through simulation that our model mimics the observation in real populations, where competition for resources increases phenotypic variability among individuals. In our model an improvement of the social environment through an increase in *b*, which was modeled as a heritable trait in itself, resulted in reduced variability.

### Estimating *b*

The key parameter in our model is the regression coefficient *b*, which comprises both direct and indirect genetic effects. In other words, *b* is heritable and can respond to selection. Application of our model requires methods to estimate *b* and its genetic components. In the following, we discuss the data requirements and propose models that could be used as a first step to estimate the average *b* and its direct and indirect genetic variance.

Our *b* connects the difference in trait values between the group mate and the focal individual at the previous time point to the target phenotype of the focal individual at the current time point. Estimating *b*, therefore, requires data on group-structured populations, where competition occurs within groups, and repeated observations on the phenotypes of the group members (i.e., time-series data).

First, to estimate the overall average level of competition, one could fit single fixed *b* for all groups, using the model

$$y_{t,i} = P_{t + 1,i} - P_{t,i} = \mu + b\left( {P_{t,j} - P_{t,i}} \right) + e_{t,i}.$$

In genetic analysis of outbred populations, interest is in the genetic (co)variances of growth and the direct and indirect effects on *b* (*A*_*GR*_, *A*_*D*_, and *A*_*I*_ in Eqs. 2 & 3). In animal and plant breeding, for example, knowledge of those parameters would indicate prospects for genetic selection against competition and variability. In outbred populations, the following mixed model may serve as starting point to estimate genetic variance components (ignoring non-genetic terms for simplicity),

$$\begin{array}{l}{\mathbf{y}}_{t,i} = \\ \mu _t + \bar b\Delta {\mathbf{y}}_{t - 1,ij} + {\mathbf{Za}}_{{{GR}}} + {\mathbf{Z}}_{{{D}},\Delta y_{t - 1,ij}}{\mathbf{a}}_{{D}} + {\mathbf{Z}}_{{{I}},\Delta y_{t - 1,ij}}{\mathbf{a}}_{{I}} + {\mathbf{e}}\end{array}$$

where matrices and vectors are in bold and scalars are in italic. **y** is a vector of phenotypic observations, with elements ***y***_*t*,*i*_ = *P*_*t*,*i*_ − *P*_*t*−1,*i*_, *μ*_*t*_ is an overall mean that may be specific to each time point. The term $\bar b\Delta {\mathbf{y}}_{t - 1,ij}$ accounts for the average competition in the population, and Δ***y***_*t*−1,*ij*_ is a vector of phenotypic differences between the group-mate and the focal individual at the previous time point, with elements Δ***y***_*t*−1,*ij*_ = *P*_*t*−1,*j*_−*P*_*t*−1,*i*_. The ***Za***_*GR*_ are the ordinary (random) additive genetic effects on growth rate. The ${\mathbf{Z}}_{{{D}},\Delta y_{t - 1,ij}}{\mathbf{a}}_{{D}}$ accounts for the direct genetic effects in *b*, where **a**_*D*_ is a vector of random direct genetic effects on *b*, and ${\mathbf{Z}}_{{{D}},\Delta y_{t - 1,ij}}$ an incidence matrix for direct effects, with elements *P*_*t*−1,*j*_−*P*_*t*−1,*i*_ in the row and column for focal individual *i*. The ${\mathbf{Z}}_{I,\Delta y_{t - 1,ij}}{\mathbf{a}}_I$ accounts for the indirect genetic effects in *b*, where ***a***_*I*_ is a vector of random indirect genetic effects on *b*, and ${\mathbf{Z}}_{I,\Delta y_{t - 1,ij}}$ is an incidence matrix for indirect effects, with elements *P*_*t*−1,*j*_−*P*_*t*−1,*i*_ in the row for the focal individual *i* and column for its group mate *j*. Hence, direct and indirect effects on *b* are so-called random regressions. Note that the above expression merely serves as starting point, and will have to be extended with non-genetic random effects, such random group effects and permanent individual effects (*E*_*p*,*GR*,*i*_ in Eq. 2). Moreover, there may be issues with the identifiability of the genetic variance components, which will depend on the family relationships within and between groups (e.g., Appendix of Bijma et al. 2007b).

When time series data are not available, which may often be the case, another approach could offer a solution. Quantitative genetic models for inherited variability can be used to estimate genetic variance in variability from records on within-family variance. Figure 4a shows that variability of sire offspring is correlated with the direct breeding value for *b* of the sire. Figure 4b shows that variability of the group mates of the offspring is correlated with the indirect breeding value for *b* of the sire. Therefore, it may be possible to capture direct and indirect effects on *b* by fitting linear mixed models to the within-family variance, and to the variance of the group mates of a family, with sire as random effect. This analysis requires an appropriate family and group structure, but not time series data. More research is needed to see how breeding values for inherited variability correlate with direct and indirect effects on *b*, and how those effects can be fully captured.

### Evidence for genetic variation in *b*

To the best of our knowledge, there are no estimates of *b* available in the literature. However, some indications for variation in *b* may come from estimates of *ψ* (psi) in so-called trait-based models of IGE (Moore et al. 1997). When data are available on multiple discrete genotypes, such as inbred lines, fixed *b* values could be estimated for each genotype, similar to the approach of Bleakley and Brodie (2009), who estimated *ψ* in guppies (Eq. 1).

This empirical study involved five inbred strains of guppies that differed genetically in their antipredator behavior. One individual from each (focal) strain was paired with three individuals from a different, unrelated strain i.e. social strain. In that way, each focal genotype was tested against different social environments. The results of the study show that the level of *ψ* differed between the focal strains and in some cases also depended on the social strain, suggesting genetic variation in *ψ*. In a similar experimental design, where the focal genotype was held constant while social groups varied, the social group effects were estimated for chemical signaling in *D. melanogaster* (Kent et al. 2008) and sexual display traits in *D. serrata* (Chenoweth et al. 2010), and both studies estimated and found variation in *ψ*.

### Implications for animal and plant breeding

Phenotypic uniformity is an important trait in animal breeding. In the pig industry, for example, it is desirable to deliver animals within a preferred range to the slaughter house, while deliveries outside that range result in penalties for the farmer (Hennessy 2005; Mulder et al. 2008). In aquaculture, fish that deviate too much from the average size are usually not sold, which reduces revenues. In addition, large size differences in fish populations stimulate competition, which reduces welfare and health of the animals. Better understanding of inherited variability, therefore, is interesting from an economic and animal welfare point of view. In plants, variability may also emerge as a commercially important trait, as some studies suggest that higher uniformity is related to higher productivity (Zhang et al. 1999; Denison et al. 2003).

There is substantial evidence of a genetic basis of variability, which has been obtained through selection experiments and by quantifying genetic variation in variability (reviewed by Hill and Mulder 2010). Recently, methods have been developed to detect QTLs that control variability, so-called vQTLs (Rönnegård and Valdar 2011; Rönnegård and Valdar 2012), and these have been found in studies of litter size in pigs (Sell-Kubiak et al. 2015), several morphological traits and days to flowering in maize (Ordas et al. 2008), and locomotor behavior in fruit flies (Ayroles et al. 2015). Furthermore, several mechanisms, resulting in vQTL effects have been proposed (Rönnegård and Valdar 2011; Rönnegård and Valdar 2012), including: epistatic gene interaction, gene-by-environmental interaction, multi-allelic additive effects underlying a QTL and scale of measurement for the observed phenotype. However, until now, variability has been studied only in relation to direct genetic effects of the focal individual. Here we considered an alternative mechanism that gives rise to genetic variation in variability, which does not only involve the genotype of the focal individual, but also a genetic effect of the social partner, and hence adds another layer to the complexity of inherited variability. The genetic contribution of the social partner is ignored in current QG models for inherited variability, which may reduce accuracy of estimated breeding values and response to selection. When traits are affected by social interactions, selection strategies that accounts for both direct and indirect genetic effects can result in higher response (for example, Griffing 1976; Muir 1996; Bijma et al. 2007b). Our findings suggest that future breeding programs aiming to reduce variability may also need to consider increasing *b*.

### Implications for evolutionary biology

In evolutionary biology, the study of canalization focuses on the absence or suppression of phenotypic variation. Hence, breeding for uniformity can be seen as an analog of the evolution of canalization. Results of our model suggest that canalization may have a social genetic component. Evolution of canalization, therefore, could also be studied in the light of the regression coefficient *b*. A better understanding of the genetic mechanisms affecting variation may also increase our understanding of the potential for evolutionary change (Flatt 2005). For example, traits may show less variability in some populations than in others, which is often attributed to low genetic variation. With canalization, however, phenotypic variation may be low while the underlying genetic variation is high, which can hinder phenotypic evolution (Flatt 2005).

Mulder et al. (2016) showed that within-nest variability of fledging weight in a natural population of Great Tit (*Parus major*) has a genetic component and is under stabilizing selection. In that study, phenotypic variability was considered either a trait of the individual, or a trait of its parents, and it was discussed how this view would change the interpretation of the genetic parameters. Here we focused at connecting differences in phenotypic variability between individuals to the level of competition, which may be useful for future studies on variability in natural populations.

Kin-selection theory predicts that individuals should interact differentially with kin vs. non-kin, because this increases their inclusive fitness (Hamilton 1964b). Together with results of our model, this prediction suggests that related individuals should show less variability. In other words, groups consisting of relatives should have higher $\bar b$ than groups of unrelated individuals. Hence, our findings suggest that canalization may partly evolve by kin-selection.

## Conclusion

We presented a quantitative genetic model in which direct and indirect genetic effects lead to inherited variability of trait values on the phenotypic level. The *b* from our model can respond to selection, and changes in *b* resulted in changes in variability, indicating the co-evolution of social interactions and inherited variability. Selection results showed that the effect of IGEs on *b* is ignored in classical mass selection, but can be partly captured by group selection on the mean or the variance. The latter also resulted in a decrease of variability. These findings suggest that we may have been overlooking an entire level of genetic variation in variability, the one due to IGEs. Genetic improvement of social effects, therefore, may be a promising route to reduce variability.

### Data archiving

The data are available from the Dryad Digital Repository: 10.5061/dryad.c3k48j0

Box 1: Direct and indirect breeding values for *b* Direct breeding value (*A*_*D*_) is the additive genetic effect of the focal individual on its own *b* and is referred to as a “resistance to competition”. Negative *A*_*D*_ would mean that the individual is sensitive to competition, while the individual with positive *A*_*D*_ is resistant to competition.

Indirect breeding value (*A*_*I*_) refers to additive genetic effect of a social partner on *b* of a focal individual. It is also referred to as “cooperativeness”. The social partner with negative *A*_*I*_ is competitive, while the one with positive *A*_*I*_ is cooperative.

Each individual, therefore, has two breeding values for *b*—one that affects their own *b* and one that affects their social partner’s *b*.

If we consider two individuals, *i* and *j*, that differ in their body size in the previous time period by 2 g, such as that *j* is the larger individual, i.e., *P*_*t*−1,*j*_ − *P*_*t*−1,*i*_ = 2 g and *P*_*t*−1,*i*_ − *P*_*t*−1,*j*_ = −2 g, then the change in phenotype for individual *i* from time *t*−1 to *t* is given as Δ*P*_*t*,*i*_ = *b*_*ij*_(*P*_*t*−1,*j*_ − *P*_*t*−1,*i*_) = 2*b*_*ij*_, and similarly Δ*P*_*t*,*j*_ = *b*_*ji*_(*P*_*t*−1,*i*_ − *P*_*t*−1,*j*_) = −2*b*_*ij*_ (Eq. 2, assuming no effect of breeding value for growth and no environmental effects).

As given in Eq. 3, $b_{ij} = \bar b + A_{{{D}},i} + A_{{{I}},j}$, where $\overline {b}$ is population parameter, negative with competition and positive with cooperation, 0 when neutral. Correspondingly, $b_{ji} = \bar b + A_{{{D}},j} + A_{{{I}},i}$.

**Competitive environment**

In the competitive environment, where, for example, $\overline b$ = −0.05, and both individuals are cooperative and resistant to competition, with breeding values of 0.03, i.e., *A*_*D*,*i*_ = *A*_*D*,*j*_ = *A*_*I*,*j*_ = *A*_*I*,*i*_ = 0.03, the change in growth for individual *i* (Δ*P*_*t*,*i*_) is 0.02 g, while Δ*P*_*t*,*j*_ = −0.02 g. However, if both individuals are competitive and sensitive to competition, *A*_*D*,*i*_ = *A*_*D*,*j*_ = *A*_*I*,*j*_ = *A*_*I*,*i*_ = −0.03, then Δ*P*_*t*,*i*_ = −0.22 g, while Δ*P*_*t*,*j*_ = 0.22 g. Hence, in a competitive environment, when both $\overline b$ and individual breeding values for *b* are negative, the larger individual grows fast, while the growth of smaller one is slowed down. Positive breeding values in a competitive environment lead to small increase in growth for the smaller individual, and a small decrease for the larger one.

For explanation on chosen values see “Simulation and Appendix C” section.

**Cooperative environment**

In the cooperative environment, where $\overline b$ is, for example, 0.05, and individuals have positive breeding values of 0.03, Δ*P*_*t*,*i*_ = 0.22 g, while Δ*P*_*t*,*j*_ = −0.22 g. If both individuals have all negative breeding values of −0.03, then Δ*P*_*t*,*i*_ = −0.02 g, while Δ*P*_*t*,*j*_ = 0.02 g. Therefore, when both $\overline b$ and individual breeding values for *b* are positive, the growth of the larger individual slows down, allowing the smaller individual to catch up. Negative breeding values in a cooperative environment lead to small increase in growth for the larger individual, and a small decrease in growth for the smaller individual.

For more scenarios and effects of combining positive and negative breeding values for *b*, see Supplementary File 1.

### Electronic supplementary material

Supplementary file 1(DOCX 17 kb)

Supplementary file 2(XLSX 29 kb)

Supplementary file 3(DOCX 1155 kb)

Supplementary file 4(DOCX 16 kb)

Supplementary file 5(DOCX 13 kb)

Supplementary file 6(DOCX 20 kb)

## Appendix A

In this appendix, explicit expressions for the expectation and the variance of the difference between the phenotypic values for individual *i* and *j* at time point *T* are derived, i.e. *E*(*P*_*T*,*i*_−*P*_*T*,*j*_|*b*_*ij*_,*b*_*ji*_) and *V*(*P*_*T*,*i*_−*P*_*T*,*j*_|*b*_*ij*_,*b*_*ji*_) respectively. By deriving these formula, given the parameters *b*_*ij*_ and *b*_*ji*_, it is possible to study the effect of these parameter values on the expectation and variance of the phenotypic difference. The derived formulae show that the expected difference is 0 if there is no initial difference at *T* = 0 (i.e. Δ*P*_0_ = 0), whereas the variance depends directly on the sum of *b*_*ij*_ and *b*_*ji*_.

The model used throughout the paper for individuals *i* and *j* is

$$P_{t,i} = P_{t - 1,i} + \mu _{{{GR}}} + A_{{{GR}},i} + E_{p,{{GR}},i} + E_{t,{{GR}},i} + b_{i,j}\left( {P_{t - 1,j} - P_{t - 1,i}} \right)$$

$$P_{t,j} = P_{t - 1,j} + \mu _{{{GR}}} + A_{{{GR}},j} + E_{p,{{GR}},j} + E_{t,{{GR}},j} + b_{j,i}\left( {P_{t - 1,i} - P_{t - 1,j}} \right)$$

Let Δ*P*_*t*_ = *P*_*t*,*i*_ − *P*_*t*,*j*_, Δ*A*_*GR*_ = *A*_*GR*,*i*_−*A*_*GR*,*j*_, Δ*E*_*p*,*GR*_ = *E*_*p*,GR,*i*_ − *E*_*p*,*GR*,*j*_ and Δ*E*_*t*,*GR*_ = *E*_*t*,*GR*,*i*_ − *E*_*t,GR*,*j*_. Then we have the recursive formula:

$$\Delta P_{t + 1} = \left( {1 - \left( {b_{ij} + b_{ji}} \right)} \right)\Delta P_t + \Delta A_{{{GR}}} + \Delta E_{p,{{GR}}} + \Delta E_{t,{{GR}}},$$

which can be written in explicit form for time *T* (in our simulations *T* = 10):

$$\Delta P_T = \lambda ^T\Delta P_0 + \mathop {\sum }\limits_{t = 0}^{T - 1} \lambda ^t\left( {\Delta A_{{{GR}}} + \Delta E_{p,{{GR}}}} \right) + \mathop {\sum }\limits_{t = 0}^{T - 1} \lambda ^t\Delta E_{t,{{GR}}}$$

where *λ* = 1−(*b*_*ij*_ + *b*_*ji*_).

Noting that the first sum is a geometric series multiplied by a constant the formula can be simplified:

$$\Delta P_T = \lambda ^T\Delta P_0 + \frac{{\lambda ^T - 1}}{{\lambda - 1}}\left( {\Delta A_{{{GR}}} + \Delta E_{p,{{GR}}}} \right) + \mathop {\sum }\limits_{t = 0}^{T - 1} \lambda ^t\Delta E_{t,{{GR}}}$$

Thus, the expected difference given the parameters *b*_*ij*_ and *b*_*ji*_ is:

$$\begin{array}{*{20}{l}} {{E}}(\Delta P_T|b_{ij},b_{ji}) & = \lambda ^T\Delta P_0 + \frac{{\lambda ^T - 1}}{{\lambda - 1}}{{E}}\left( {\Delta A_{{{GR}}} + \Delta E_{p,{{GR}}}} \right)\\ &\quad + \mathop {\sum }\limits_{t = 0}^{T - 1} \lambda ^t{{E}}(\Delta E_{t,{{GR}}})\\ & = \lambda ^T\Delta P_0 + \frac{{\lambda ^T - 1}}{{\lambda - 1}}{{E}}\left( {\Delta A_{{{GR}}} + \Delta E_{p,{{GR}}}} \right)\\ & \quad + \frac{{\lambda ^T - 1}}{{\lambda - 1}}{{E}}(\Delta E_{t,GR}) \end{array}$$

and is equal to 0 for Δ*P*_0_ = 0.

The variance of the difference in phenotypes given the parameters *b*_*ij*_ and *b*_*ji*_ is:

$$\begin{array}{*{20}{l}}V\left( {\Delta P_T{\mathrm{|}}b_{ij},b_{ji}} \right) & = \left( {\frac{{\lambda ^T - 1}}{{\lambda - 1}}} \right)^2V\left( {\Delta A_{{{GR}}} + \Delta E_{p,{{GR}}}} \right)\\ & \quad + \mathop {\sum }\limits_{t = 0}^{T - 1} \left( {\lambda ^t} \right)^2V\left( {\Delta E_{t,{{GR}}}} \right)\\ & = \left( {\frac{{\lambda ^T - 1}}{{\lambda - 1}}} \right)^2V\left( {\Delta A_{GR} + \Delta E_{p,GR}} \right)\\ & \quad + \frac{{\lambda ^{2T} - 1}}{{\lambda - 1}}V\left( {\Delta E_{t,{{GR}}}} \right) \end{array}$$

For the special case $b_{ij}^{} + b_{ji}^{} = 0$, *V*(Δ*P*_*T*_|*b*_*ij*_,*b*_*ji*_) = *T*^2^ × *V*(Δ*A*_*GR*_ + Δ*E*_*p*,*GR*_) + *T* × *V*(Δ*E*_*t*,*GR*_).

Furthermore, $\frac{{\lambda ^T - 1}}{{\lambda - 1}} < T$ and $\frac{{\lambda ^{2T} - 1}}{{\lambda - 1}} < T$ for *λ* < 1, and for *λ* > 1 we have $\frac{{\lambda ^T - 1}}{{\lambda - 1}} > T$ and $\frac{{\lambda ^{2T} - 1}}{{\lambda - 1}} > T$. Recall that *λ* = 1 − (*b*_*ij*_ + *b*_*ji*_). Thus, the variance of the phenotypic difference will be smaller than the variance for a model without social interaction effects (i.e., *P*_*t*,*i*_ = *P*_*t*−1,*i*_ + *μ*_*GR*_ + *A*_*GR*,*i*_ + *E*_*p*,*GR*,*i*_ + *E*_*t*,*GR*,*i*_) if $b_{ij} + b_{ji} > 0$, and larger if $b_{ij} + b_{ji} < 0$.

### Matrix version of the model

In the following part of the appendix, it is shown how the model can be written in matrix form and the variance of the individual phenotypes (given *b*_*ij*_ and *b*_*ji*_) can be derived. Hence, an advantage of writing the model in matrix form is that we can derive an expression for the variance of the individual phenotypes, whereas in the previous derivations the variance of the phenotypic difference was derived. Furthermore, by studying the eigenvalues of the matrices in the model, the sensitivity to stochastic environmental effects can be assessed. The matrix notation can also be a tool to simplify computations in simulation studies.

An important result derived below is that the phenotypic values at the final time point *T* are sensitive to the simulated environmental variables (error terms) if *b*_*ij*_ + *b*_*ji*_ < 0.

Equation 2 can be written in matrix form for the two individuals *i* and *j* simultaneously as:

$$\begin{array}{l}P_{t + 1} = BP_t + \Delta + \varepsilon _t\\ P_t = \left( {\begin{array}{*{20}{c}} {P_{t,i}} \\ {P_{t,j}} \end{array}} \right)\\ B = \left( {\begin{array}{*{20}{c}} {1 - b_{ij}} & {b_{ij}} \\ {b_{ji}} & {1 - b_{ji}} \end{array}} \right)\\ \Delta = \left( {\begin{array}{*{20}{c}} {\mu _{{{GR}}} + A_{{{GR}},i} + E_{p,{{GR}},i}} \\ {\mu _{{{GR}}} + A_{{{GR}},j} + E_{p,{{GR}},j}} \end{array}} \right)\\ \varepsilon _t = \left( {\begin{array}{*{20}{c}} {E_{t,GR,i}} \\ {E_{t,{{GR}},j}} \end{array}} \right)\end{array}$$

with expectation and variance for Δ and *ε*_*t*_

$$\begin{array}{l}E\left( {\mathrm{\Delta }} \right) = \left( {\begin{array}{*{20}{c}} {\mu _{{{GR}}}} \\ {\mu _{{{GR}}}} \end{array}} \right),V\left( {\mathrm{\Delta }} \right) = \left( {\sigma _{A_{{{GR}}}}^2 + \sigma _{E_{p,{{GR}}}}^2} \right){\boldsymbol{I}}\\ E\left( {\varepsilon _t} \right) = \left( {\begin{array}{*{20}{c}} 0 \\ 0 \end{array}} \right),V\left( {\varepsilon _t} \right) = \sigma _\varepsilon ^2{\boldsymbol{I}}\end{array}$$

In the formula for *V*(Δ), the two individuals are assumed to be unrelated.

At time *T* (with *T* = 10 in our simulations) we then have

$$P_T = B^TP_0 + \mathop {\sum }\limits_{k = 0}^{T - 1} B^k\Delta + \mathop {\sum }\limits_{k = 0}^{T - 1} B^k\varepsilon _k$$

In the simulations, the initial phenotypes were set to zero, i.e. *P*_0_ = 0. Hence, the first term can be ignored and we can focus on the second and third terms, i.e. the sums, in the above formula.

Let *B* = ΓΛΓ^−1^ be the spectral decomposition of *B*, with eigenvalues $\lambda _1^{}$ = 1 and $\lambda _2 = 1 - (b_{ij} + b_{ji})$.

One can note that the phenotypes at time *T*, i.e., *P*_*T*_, will be sensitive to the simulated environmental variables (error terms) if *b*_*ij*_ + *b*_*ji*_ < 0 because the dominating eigenvalue will then be greater than 1.

Furthermore, the first sum in the above formula is a geometric series and can be written as:

$$\mathop {\sum }\limits_{k = 0}^{T - 1} B^k\Delta = {\mathrm{\Gamma }}\left( {\begin{array}{*{20}{c}} T & 0 \\ 0 & {\frac{{\lambda _2^T - 1}}{{\lambda _2^{} - 1}}} \end{array}} \right){\mathrm{\Gamma }}^{ - 1}\Delta$$

with expectation

$$E\left( {\mathop {\sum }\limits_{k = 0}^{T - 1} B^k\Delta } \right) = {\mathrm{\Gamma }}\left( {\begin{array}{*{20}{c}} T & 0 \\ 0 & {\frac{{\lambda _2^T - 1}}{{\lambda _2^{} - 1}}} \end{array}} \right){\mathrm{\Gamma }}^{ - 1}E(\Delta ).$$

The variance of the sum is

$$V\left( {\mathop {\sum }\limits_{k = 0}^{T - 1} B^k\Delta } \right) = {\mathrm{\Gamma }}\left( {\begin{array}{*{20}{c}} T & 0 \\ 0 & {\frac{{\lambda _2^T - 1}}{{\lambda _2^{} - 1}}} \end{array}} \right){\mathrm{\Gamma }}^{ - 1}\left( {{\mathrm{\Gamma }}^{ - 1}} \right)^\prime \left( {\begin{array}{*{20}{c}} T & 0 \\ 0 & {\frac{{\lambda _2^T - 1}}{{\lambda _2^{} - 1}}} \end{array}} \right){\mathrm{\Gamma }}^\prime V(\Delta ).$$

The variance of the second sum is

$$V\left( {\mathop {\sum }\limits_{k = 0}^{T - 1} B^k\varepsilon _k} \right) = \mathop {\sum }\limits_{k = 0}^{T - 1} B^k\left( {B^k} \right)^\prime V(\varepsilon _k)$$

with

$$V\left( {\varepsilon _k} \right) = I\sigma _\varepsilon ^2$$

and

$$\mathop {\sum }\limits_{k = 0}^{T - 1} B^k\left( {B^k} \right)^\prime = {\mathrm{\Gamma }}\left( {\begin{array}{*{20}{c}} {A_{11}T} & {A_{12}\frac{{\lambda _2^T - 1}}{{\lambda _2^{} - 1}}} \\ {A_{21}\frac{{\lambda _2^T - 1}}{{\lambda _2^{} - 1}}} & {A_{22}\frac{{\lambda _2^{2T} - 1}}{{\lambda _2^2 - 1}}} \end{array}} \right){\mathrm{\Gamma }}^\prime,$$

where *A*_*kl*_ is the element on row *k* and column *l* in the matrix *A* = Γ^−1^(Γ^−1^).^′^

Thus,

$$V\left( {\mathop {\sum }\limits_{k = 0}^{T - 1} B^k\varepsilon _k} \right) = {\mathrm{\Gamma }}\left( {\begin{array}{*{20}{c}} {A_{11}T} & {A_{12}\frac{{\lambda _2^T - 1}}{{\lambda _2^{} - 1}}} \\ {A_{21}\frac{{\lambda _2^T - 1}}{{\lambda _2^{} - 1}}} & {A_{22}\frac{{\lambda _2^{2T} - 1}}{{\lambda _2^2 - 1}}} \end{array}} \right){\mathrm{\Gamma }}^\prime \sigma _\varepsilon ^2$$

with $$A_{11} = A_{22} = \frac{{2(b_{ij}^2 + b_{ji}^2)}}{{(b_{ij}^{} + b_{ji}^{})^2}}$$ and $$A_{12} = A_{21} = \frac{{b_{ji} - b_{ij}}}{{\left( {b_{ij} + b_{ji}} \right)^2}}\sqrt {2(b_{ij}^2 + b_{ji}^2)}$$.

Using the matrix of eigenvectors

$${\mathrm{\Gamma }} = \left( {\begin{array}{*{20}{c}} {\sqrt {\frac{1}{2}} } & {\frac{{b_{ij}}}{{\sqrt {b_{ij}^2 + b_{ji}^2} }}} \\ {\sqrt {\frac{1}{2}} } & {\frac{{ - b_{ji}}}{{\sqrt {b_{ij}^2 + b_{ji}^2} }}} \end{array}} \right)$$

and its inverse

$${\mathrm{\Gamma }}^{ - 1} = \left( {\begin{array}{*{20}{c}} {\frac{{b_{ji}}}{{\sqrt {b_{ij}^2 + b_{ji}^2} }}} & {\frac{{b_{ij}}}{{\sqrt {b_{ij}^2 + b_{ji}^2} }}} \\ {\sqrt {\frac{1}{2}} } & { - \sqrt {\frac{1}{2}} } \end{array}} \right) \times \frac{{\sqrt {2(b_{ij}^2 + b_{ji}^2)} }}{{b_{ij}^{} + b_{ji}^{}}}$$

Using the relationship $$V\left( {P_{T,i} - P_{T,j}} \right) = \left( {\begin{array}{*{20}{c}} 1 & { - 1} \end{array}} \right)V(P_T)\left( {\begin{array}{*{20}{c}} 1 \\ { - 1} \end{array}} \right)$$ and applying several steps of straight-forward derivations, the same expression for the variance of the phenotypic difference is derived as above

$$V\left( {P_{T,i} - P_{T,j}|b_{ij},b_{ji}} \right) = 2D_1\left( {\sigma _{A_{{{GR}}}}^2 + \sigma _{E_{p,{{GR}}}}^2} \right) + 2D_2\sigma _\varepsilon ^2$$

with $D_1 = \left( {\frac{{\lambda _2^T - 1}}{{\lambda _2^{} - 1}}} \right)^2$ and $D_2 = \frac{{\lambda _2^{2T} - 1}}{{\lambda _2^2 - 1}}$ for $b_{ij}^{} + b_{ji}^{} \ne 0$, while for $b_{ij}^{} + b_{ji}^{} = 0$ we have *D*_1_ = *T*^2^ and *D*_2_ = *T*. Hence, we have derived the variance for the difference between phenotypes of two group members, i.e. *V*(*P*_*T*,*i*_−*P*_*T*,*j*_|*b*_*ij*_,*b*_*ji*_). Furthermore, the above derivations give an explicit expression for the variances (and covariances) of the individual phenotypes for the two group members, i.e., $$V\left( {\left( {\begin{array}{*{20}{c}} {P_{T,i}} \\ {P_{T,j}} \end{array}} \right)|b_{ij},b_{ji}} \right)$$. Using this expression one can assess how the variances (and covariances) at time T depend on the values of $b_{ij}^{}$ and $b_{ji}^{}$.

## Appendix B

### Larger group size

We presented a model for interaction between 2 individuals. To accommodate interactions among more individuals, Eq. 2 could be extended as follows

5 $$\begin{array}{*{20}{l}} P_{t,i} - P_{t - 1,i} = & \mu _{{{GR}}} + A_{{{GR}},i} + E_{p,{{GR}},i} + E_{t,{{GR}},i}\\ & + \mathop {\sum }\limits_{j = 1}^n b_{ij}\left( {P_{t - 1,j} - P_{t - 1,i}} \right)\\ \end{array}$$

so that the effect of a difference in trait value between a group mate *j* and focal individual *i* is summed over the *n* group mates.

## Appendix C

### Simulation description

#### Population structure

Monte Carlo simulations were conducted using the R software (R Development Core Team 2011). We first simulated a base population of 100 sires and 10,000 dams, all unrelated. Each animal was assigned a breeding value for growth rate and direct and indirect breeding value for *b*, drawn from a multivariate normal distribution. Next, we created the offspring population by mating each sire with 100 randomly chosen dams. Each sire had 100 offspring. The total number of individuals in the offspring population was 10,000. The breeding values for growth rate and direct and indirect breeding values for *b* in the offspring population were simulated as the average breeding value of sire and dam, plus a Mendelian sampling term drawn from $$N\left( {\left[ {\begin{array}{*{20}{c}} 0 \\ 0 \\ 0 \end{array}} \right]} \right.,\,\frac{1}{2}\left. {\left[ {\begin{array}{*{20}{c}} {{\mathrm{\sigma }}_{{{A}}_{{{GR}}}}^2} & 0 & 0 \\ 0 & {{\mathrm{\sigma }}_{{{A}}_{{D}}}^2} & 0 \\ 0 & 0 & {{\mathrm{\sigma }}_{{{A}}_{{I}}}^2} \end{array}} \right]} \right)$$. Each offspring was also given the permanent and temporary environmental effect on body weight, as well as direct and indirect environmental effects on *b*. These were sampled from $$N\,\left. {\left( {\left[ {\begin{array}{*{20}{c}} 0 \\ 0 \\ 0 \\ 0 \end{array}} \right]}, \right.\left[ {\begin{array}{*{20}{c}} {{{\sigma }}_{{{E}}_{{{p}},{{GR}}}}^2} & 0 & 0 & 0 \\ 0 & {{{\sigma }}_{{{E}}_{{{t}},{{GR}}}}^2} & 0 & 0 \\ 0 & 0 & {{{\sigma }}_{{{E}}_{{D}}}^2} & 0 \\ 0 & 0 & 0 & {{{\sigma }}_{{{E}}_{{I}}}^2} \end{array}} \right]} \right)$$. All genetic and environmental covariances were set to zero. Individuals from offspring population were randomly assigned to groups of 2 members, creating 5000 groups in total. Finally, growth curves of individuals were simulated by creating phenotypes for 10 time points using Eq. 2. Therefore, each individual had repeated observations.

For the selection part, we used a simulated population of individuals (offspring population) as explained in previous paragraph to be the base population. Three types of selections were performed, individual and 2 group selections, using phenotypes from the last time point i.e. time point 10. Individual selection was made on body weight, by selecting 11% of best individuals. First group selection was made on average body weight of the pair making up a group, while second group selection was performed on the squared difference in body weight within a pair, i.e., the variance among the two group mates. In both group selections 11% of best groups were selected. Selections were performed for 10 generations. To maintain the same number of individuals through selection (10,000), sex ratio and mating was performed differently in the selection generations compared to the base. Sex was randomly assigned to 1100 selected individuals in 1 male: 10 females probability, and 1 male was mated with 10 randomly chosen females. The genetic and environmental values of offspring, group assignment and phenotype construction was done in the same manner as described in the previous paragraph.

#### Parameters

Table 2 contains parameters used in the simulation. In farmed aquaculture species, for example Nile tilapia, fish weights around 10 g (g) when it is first stocked into the pond, and between 100 and 200 g at the end of the growth period. To connect our results somewhat to aquaculture species, we have set 10 g as a mean starting weight and assumed that in every time period individuals gain on average 10 g. Therefore, mean growth rate, (μ_*GR*_) was 10 g. The genetic standard deviation of growth rate (${{\sigma }}_{{{A}}_{{{GR}}}}^{}$) was 10% of μ_*GR*_, which was 1 g, therefore ${{\sigma }}_{{{A}}_{{{GR}}}}^2$ was 1 g^2^.

The range of $\bar b$ values was from −0.08 to 0.08. Standard deviation of $\bar b$ was set as 60% of $\bar b$= −0.05. Therefore, standard deviations of genetic and environmental components of *b* were calculated as $\sqrt {\sigma _{A_{\rm{D}}}^2 + \sigma _{A_{{I}}}^2 + \sigma _{E_{\rm{D}}}^2 + \sigma _{E_{{I}}}^2} = 0.6\bar b$, and since all variances were assumed equal, each of them had value of 0.225 × 10^−3^ (Table 2).

Repeatability was set to 0.7 and heritability of growth rate to 0.5, in absence of social interactions (*b* = 0). Phenotypic variance was calculated as $\sigma _P^2$=${{\sigma }}_{{{A}}_{{{GR}}}}^2{\mathrm{/}}h^2$ and was equal to 2 g^2^, permanent environmental effect on growth (${{\sigma }}_{{{E}}_{{\mathrm{p}},{{GR}}}}^2$) as 0.2$\sigma _P^2$= 0.4 g^2^ and temporary environmental effect ($\sigma _{{{E}}_{{\mathrm{t}},{{GR}}}}^2$) as 0.3$\sigma _P^2$= 0.6 g^2^ (Table 2).

Five simulated scenarios were based on 5 different values of $\overline b$ (Table 2). For the selection part, only a base population was used with $\bar b$ of −0.08 to test the effect of selection on variability.
