# Supplementary material for: Modelling the co-evolution of indirect genetic effects and inherited variability
Source: Heredity (Edinb). 2018 Mar 28;121(6):631–47. doi: 10.1038/s41437-018-0068-z (PMC6221879; doi:10.1038/s41437-018-0068-z)
Supplement: Supplementary file 1 — (DOCX 17 kb) [file 41437_2018_68_MOESM1_ESM.docx]

**Supplementary file 1**

Supplementary file 1 consists of three tables which show growth of two individuals, *i* and *j*, between two time points (${\Delta P}_{t,i}$ and ${\Delta P}_{t,j}$), due to effect of *b x difference in body weight*, where *b* for individual *i* is $b_{ij}=\bar{b}+A_{D,i}+A_{I,j}$, and *b* for individual *j* is $b_{ji}=\bar{b}+A_{D,j}+A_{I,i}.$ $A_{D}$ and $A_{I}$ are direct and indirect breeding values for *b*, which take value of 0.03 or -0.03. The difference in body weight is given by $P_{t-1,j}-P_{t-1,i}=2 g$ and $P_{t-1,i}-P_{t-1,j}=-2 g$. In other words, individual *j* is larger than *i* in the previous time point, by 2 grams.

The $\bar{b}$is population parameter, negative with competition and positive with cooperation, 0 when neutral. Table S1 presents effect of *b x difference in body weight* on change in body weight of the focal individual when$\bar{b}$= 0 (Table S1), when $\bar{b}$= - 0.05 (Table S2), and when $\bar{b}$= 0.05 (Table S3).

The Supplementary file 1 is related to Box 1 in the main text.

| **Table S1**. Effect of *b x difference in body weight* on change in body weight of the focal individual when$\bar{b}$= 0 (no competition or cooperation) | | | | | |
| --- | --- | --- | --- | --- | --- |
| $P_{t-1,j}-P_{t-1,i}=2 g$ | ${\Delta P}_{t,i}$ | | $P_{t-1,i}-P_{t-1,j}=-2 g$ | ${\Delta P}_{t,j}$ | |
| Focal individual *i* | Social partner *j* | | Focal individual *j* | Social partner *i* | |
|  | $A_{I,j}$= 0.03 | $A_{I,j}$= - 0.03 |  | $A_{I,i}$= 0.03 | $A_{I,i}$= - 0.03 |
| $A_{D,i}$= 0.03 | 0.12 g | 0 g | $A_{D,j}$= 0.03 | - 0.12 g | 0 g |
| $A_{D,i}$= - 0.03 | 0 g | - 0.12 g | $A_{D,j}$= - 0.03 | 0 g | 0.12 g |
|  |  |  |  |  |  |
| **Table S2**. Effect of *b x difference in body weight* on change in body weight of the focal individual when $\bar{b}$=  - 0.05 (competition) | | | | | |
| $P_{t-1,j}-P_{t-1,i}=2 g$ | ${\Delta P}_{t,i}$ | | $P_{t-1,i}-P_{t-1,j}=-2 g$ | ${\Delta P}_{t,j}$ | |
| Focal individual *i* | Social partner *j* | | Focal individual *j* | Social partner *i* | |
|  | $A_{I,j}$= 0.03 | $A_{I,j}$= - 0.03 |  | $A_{I,i}$= 0.03 | $A_{I,i}$= - 0.03 |
| $A_{D,i}$= 0.03 | 0.02 g | -0.1 g | $A_{D,j}$= 0.03 | -0.02 g | 0.1 g |
| $A_{D,i}$= - 0.03 | -0.1 g | -0.22 g | $A_{D,j}$= - 0.03 | 0.1 g | 0.22 g |
|  |  |  |  |  |  |
| **Table S3**. Effect of *b x difference in body weight* on change in body weight of the focal individual when$\bar{b}$= 0.05 (cooperation) | | | | | |
| $P_{t-1,j}-P_{t-1,i}=2 g$ | ${\Delta P}_{t,i}$ | | $P_{t-1,i}-P_{t-1,j}=-2$ $g$ | ${\Delta P}_{t,j}$ | |
| Focal individual *i* | Social partner *j* | | Focal individual *j* | Social partner *i* | |
|  | $A_{I,j}$= 0.03 | $A_{I,j}$= - 0.03 |  | $A_{I,i}$= 0.03 | $A_{I,i}$= - 0.03 |
| $A_{D,i}$= 0.03 | 0.22 g | 0.1 g | $A_{D,j}$= 0.03 g | -0.22 g | -0.01 g |
| $A_{D,i}$= - 0.03 | 0.1 g | -0.02 g | $A_{D,j}$= - 0.03 g | -0.01 g | 0.02 g |
