# Supplementary material for: Modelling the co-evolution of indirect genetic effects and inherited variability
Source: Heredity (Edinb). 2018 Mar 28;121(6):631–47. doi: 10.1038/s41437-018-0068-z (PMC6221879; doi:10.1038/s41437-018-0068-z)

**Supplementary file 3**

Supplementary file 3 contains figures S1, S2, and S3. These figures present growth curves of two group mates for scenarios where $\bar{b}$= -0.05 (Figure S1), $\bar{b}$= 0 (Figure S2), and $\bar{b}$= 0.05 (Figure S3).

Scenario 2, $\bar{b}$= -0.05

**Figure S1**. Growth curves of two group mates (one larger than the other) that have lowest sum of *b*’s (A); the initially larger individual has negative *b*, the smaller one has positive *b* (B); the initially larger individual has positive *b*, the smaller one has negative *b* (C); lowest sum of *b*’s (D), for scenario 2. Each panel shows one typical replicate.


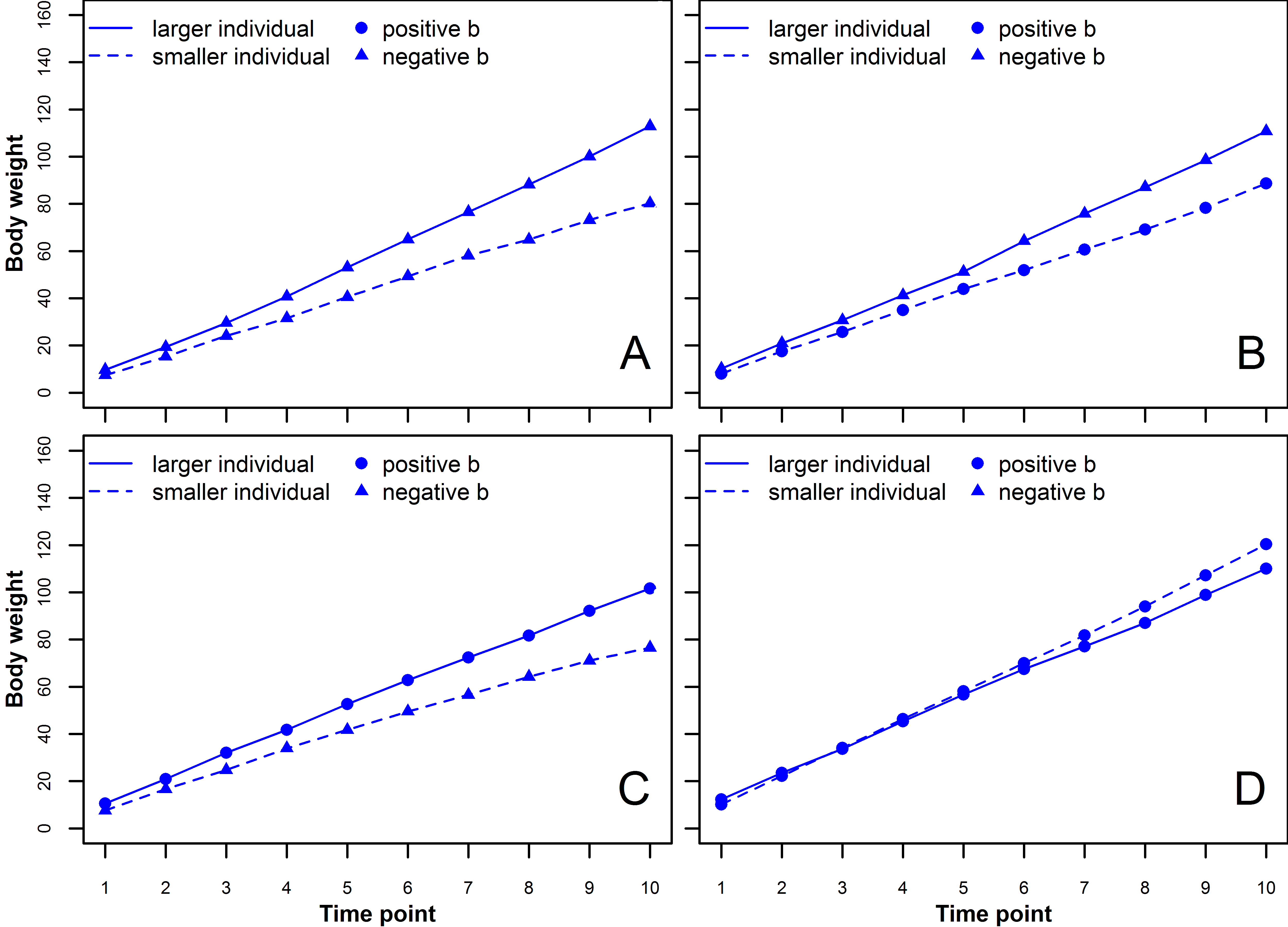


Scenario 3, $\bar{b}$= 0

**Figure S2**. Growth curves of two group mates (one larger than the other) that have lowest sum of *b*’s (A); the initially larger individual has negative *b*, the smaller one has positive *b* (B); the initially larger individual has positive *b*, the smaller one has negative *b* (C); lowest sum of *b*’s (D), for scenario 3. Each panel shows one typical replicate.


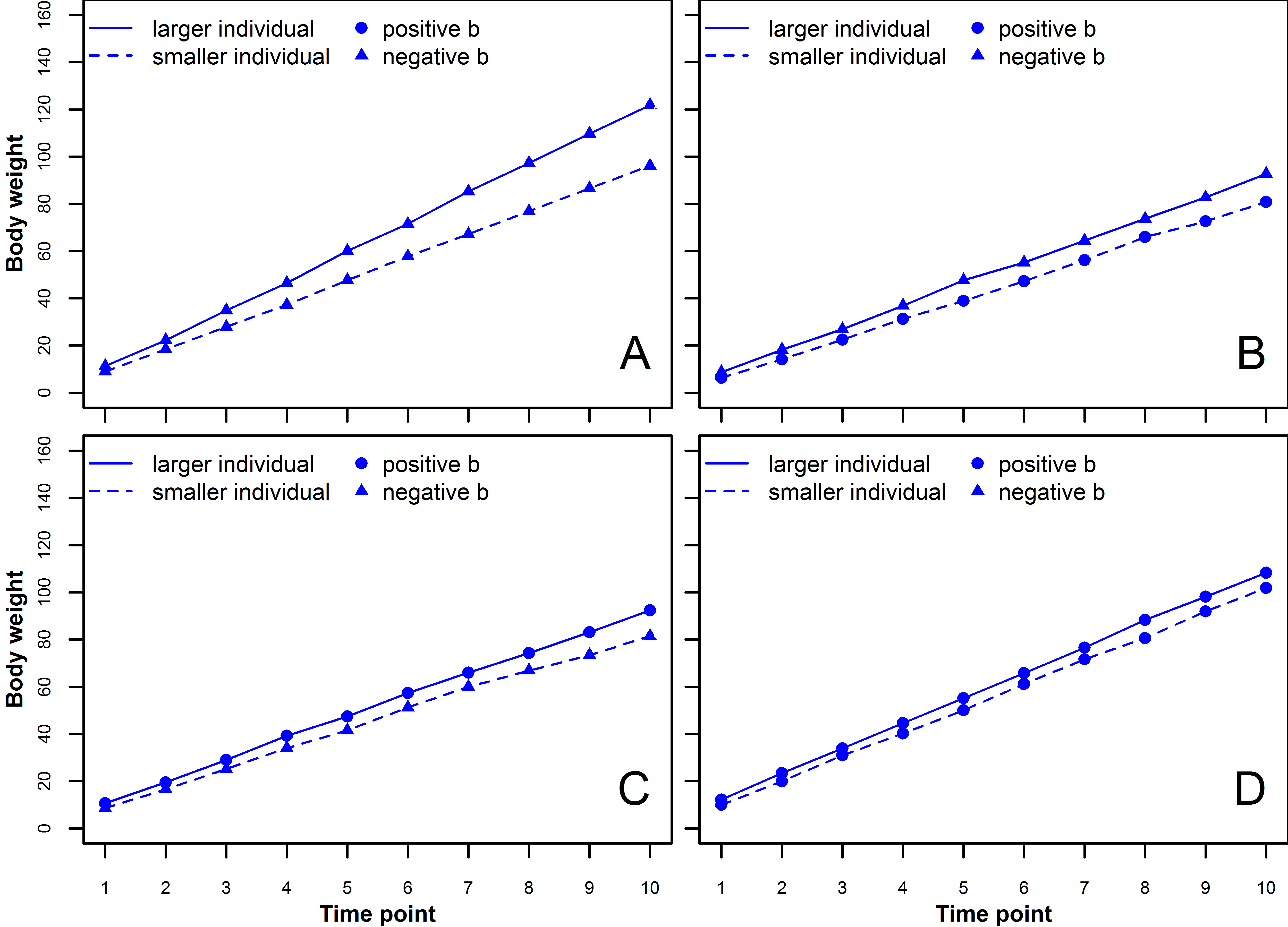


Scenario 4, $\bar{b}$= 0.05

**Figure S3**. Growth curves of two group mates (one larger than the other) that have lowest sum of b’s (A); the initially larger individual has negative b, the smaller one has positive b (B); the initially larger individual has positive b, the smaller one has negative b (C); lowest sum of b’s (D), for scenario 4. Each panel shows one typical replicate.


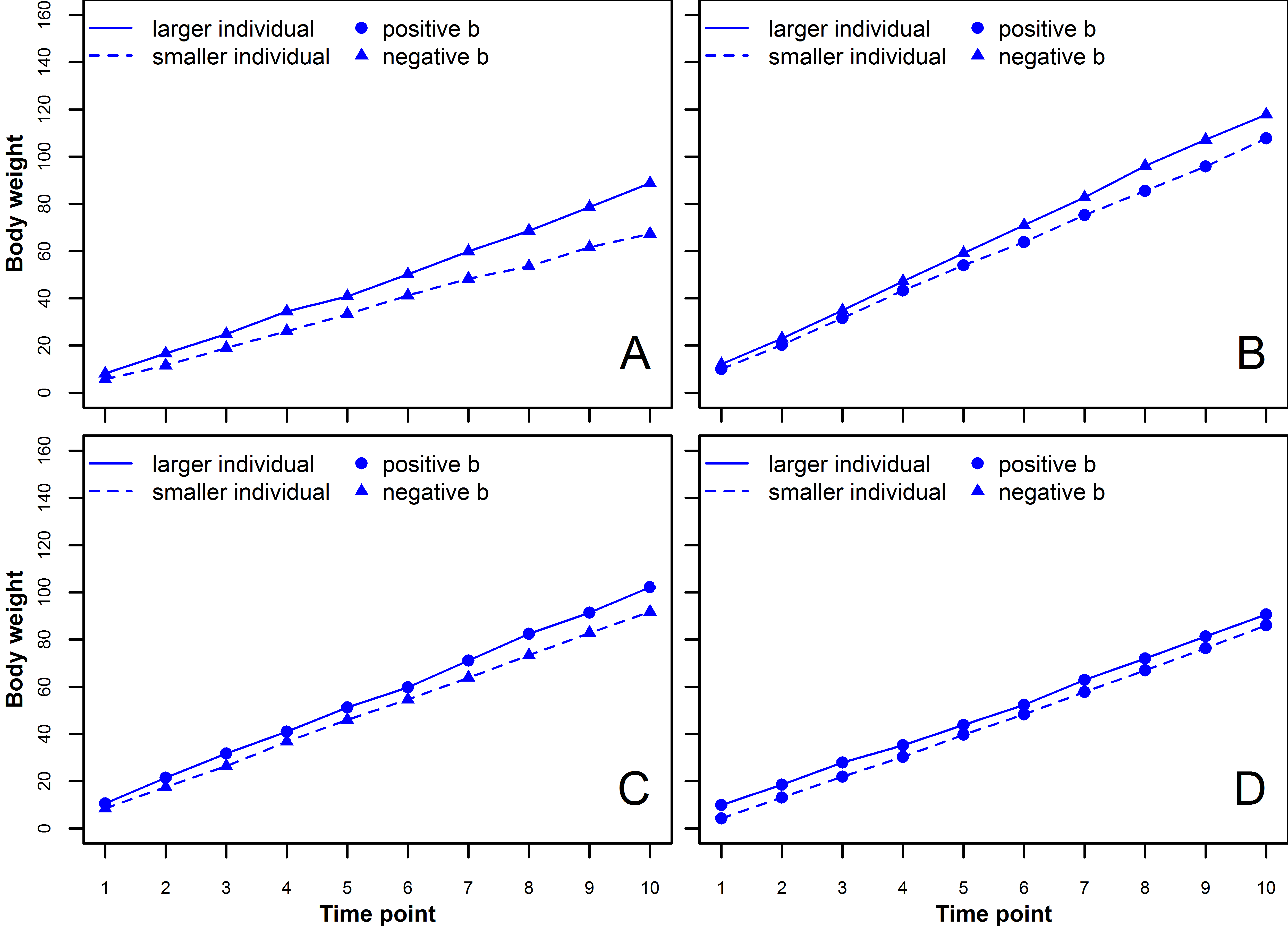

Supplement: Supplementary file 3 — (DOCX 1155 kb) [file 41437_2018_68_MOESM3_ESM.docx]
