# Supplementary material for: Modelling the co-evolution of indirect genetic effects and inherited variability
Source: Heredity (Edinb). 2018 Mar 28;121(6):631–47. doi: 10.1038/s41437-018-0068-z (PMC6221879; doi:10.1038/s41437-018-0068-z)
Supplement: Supplementary file 4 — (DOCX 16 kb) [file 41437_2018_68_MOESM4_ESM.docx]

**Supplementary file 4**

Supplementary file 4 consists of Table S4 which contains maximum and minimum sum of group mates *b*’s, averaged over 100 scenarios and Table S5 which contains *b* values of both individuals in a group, sum of their *b*’s and average squared difference of their body weights for Figure 3 in the main text and Figures S2-S4 in Supplementary file 3.

| **Table S4**. Maximum and minimum sum of *b*’s for two individuals in a group, averaged over 100 replicates | | | | | |
| --- | --- | --- | --- | --- | --- |
| Scenario | 1 | 2 | 3 | 4 | 5 |
| Min | -0.32 (0.01) | -0.26 (0.01) | -0.16 (0.01) | -0.06 (0.01) | 0.003 (0.01) |
| Max | -0.005 (0.01) | 0.05 (0.01) | 0.15 (0.01) | 0.25 (0.01) | 0.31 (0.01) |

| **Table S5**. Values of *b*, sum of *b*’s and squared difference of two individuals in a group | | | | |
| --- | --- | --- | --- | --- |
| Scenario 1 (Figure 3) | | | | |
| Panel | *b* | | Sum of *b*’s | Squared difference |
|  | Larger individual | *Smaller individual* |  |  |
| A | -0.118 | -0.127 | -0.245 | 1594.50 |
| B | -0.092 | 0.005 | -0.087 | 750.66 |
| C | 0.001 | -0.096 | -0.095 | 518.401 |
| D | 0.001 | 0.004 | 0.005 | 191.93 |
| Scenario 2 (Figure S2) | | | | |
| A | -0.111 | -0.081 | -0.192 | 1064.71 |
| B | -0.025 | 0.025 | 0.00 | 487.38 |
| C | 0.009 | -0.116 | -0.107 | 627.77 |
| D | 0.002 | 0.025 | 0.027 | 105.95 |
| Scenario 3 (Figure S3) | | | | |
| A | -0.042 | -0.042 | -0.084 | 660.36 |
| B | -0.002 | 0.010 | -0.008 | 141.03 |
| C | 0.010 | -0.077 | -0.067 | 117.95 |
| D | 0.079 | 0.049 | 0.128 | 42.35 |
| Scenario 4 (Figure S4) | | | | |
| A | -0.042 | -0.024 | -0.066 | 458.72 |
| B | -0.006 | 0.094 | 0.088 | 101.43 |
| C | 0.028 | -0.016 | -0.012 | 107.03 |
| D | 0.127 | 0.102 | 0.229 | 21.32 |
| Figure 5 (Figure 3) | | | | |
| A | -0.004 | -0.019 | -0.023 | 348.48 |
| B | -0.021 | 0.064 | 0.085 | 136.38 |
| C | 0.060 | -0.013 | 0.073 | 57.96 |
| D | 0.139 | 0.158 | 0.297 | 8.40 |
