# Supplementary material for: Modelling the co-evolution of indirect genetic effects and inherited variability
Source: Heredity (Edinb). 2018 Mar 28;121(6):631–47. doi: 10.1038/s41437-018-0068-z (PMC6221879; doi:10.1038/s41437-018-0068-z)
Supplement: Supplementary file 5 — (DOCX 13 kb) [file 41437_2018_68_MOESM5_ESM.docx]

**Supplementary file 5**

Supplementary file 5 consists of Table S6 which contains correlations between 1) direct breeding value of sires for *b* ($A_{D}$) and variability of body weight of their offspring ( ${VarP}_{off})$2) $A_{D}$ of sires and variability of body weight of group mates of sire offspring (${VarP}_{gm})$ 3) indirect breeding value of sires for *b* ($A_{I})$ and variability of body weight of their offspring 4) $A_{I}$ of sires and variability of body weight of group mates of sire offspring, for five scenarios averaged over 100 replicates with standard errors in parentheses. The table is related to Figure 4 in the main text.

| **Table S6**. Correlations | | | | |
| --- | --- | --- | --- | --- |
| Scenario | $A_{D}-$ ${VarP}_{off}$ | $A_{D}-$ ${VarP}_{gm}$ | $A_{I}-$ ${VarP}_{off}$ | $A_{I}-$ ${VarP}_{gm}$ |
| 1 | -0.54 (0.08) | -0.11 (0.09) | -0.10 (0.1) | -0.52 (0.06) |
| 2 | -0.52 (0.08) | -0.08 (0.09) | -0.07 (0.1) | -0.50 (0.07) |
| 3 | -0.43 (0.08) | -0.02 (0.09) | 0.00 (0.1) | -0.42 (0.09) |
| 4 | -0.28 (0.09) | 0.02 (0.09) | 0.04 (0.1) | -0.30 (0.08) |
| 5 | -0.21 (0.09) | 0.04 (0.09) | 0.04 (0.1) | -0.23 (0.09) |
