# Supplementary material for: Modelling the co-evolution of indirect genetic effects and inherited variability
Source: Heredity (Edinb). 2018 Mar 28;121(6):631–47. doi: 10.1038/s41437-018-0068-z (PMC6221879; doi:10.1038/s41437-018-0068-z)
Supplement: Supplementary file 6 — (DOCX 20 kb) [file 41437_2018_68_MOESM6_ESM.docx]

**Supplementary file 6**

Supplementary file 6 consists of 3 tables which contain average within-group variability, average $A_{D}$, average $A_{I}$, average $A_{GR}$, average *b*, and average body weight for base and 10 generation of selection on individual body weight (Table S7), average body weight of two individuals in a group (Table S8), and variance body weight of two individuals in a group (Table S9), averaged over 100 replicates and with standard errors between parentheses. The tables are related to Figure 5 in the main text.

| **Table S7**. Individual selection on body weight | | | | | | |
| --- | --- | --- | --- | --- | --- | --- |
|  | | | | | | |
| Generation | Variability | Average $A_{D}$ | Average $A_{I}$ | Average $A_{GR}$ | Average *b* | Average body weight |
|  | | | | | | |
| base | 376.89 (29.15) | -0.00006 (0.0003) | -0.00002 (0.0003) | 0.01 (0.002) | -0.08 (0.0003) | 100.1 (0.38) |
| 1 | 362.70 (18.82) | -0.0031 (0.001) | -0.0008 (0.001) | 1.25 (0.08) | -0.08 (0.002) | 112.48 (0.81) |
| 2 | 368.87 (23.54) | -0.0059 (0.001) | -0.001 (0.002) | 2.35 (0.10) | -0.08 (0.002) | 123.53 (1.03) |
| 3 | 386.93 (28.01) | -0.0087 (0.002) | -0.001 (0.002) | 3.41 (0.12) | -0.09 (0.003) | 134.09 (1.16) |
| 4 | 406.13 (32.79) | -0.0115 (0.002) | -0.0011 (0.002) | 4.44 (0.13) | -0.09 (0.004) | 144.42 (1.34) |
| 5 | 432.55 (40.38) | -0.0141 (0.002) | -0.0012 (0.003) | 5.48 (0.15) | -0.10 (0.004) | 154.75 (1.49) |
| 6 | 455.99 (42.47) | -0.0169 (0.003) | -0.0013 (0.003) | 6.50 (0.17) | -0.10 (0.004) | 165.04 (1.67) |
| 7 | 483.93 (49.42) | -0.0196 (0.003) | -0.0014 (0.003) | 7.53 (0.17) | -0.10 (0.005) | 175.26 (1.78) |
| 8 | 512.26 (55.38) | -0.0224 (0.003) | -0.0014 (0.004) | 8.54 (0.19) | -0.10 (0.005) | 185.40 (1.94) |
| 9 | 546.22 (62.66) | -0.0252 (0.003) | -0.0015 (0.004) | 9.56 (0.21) | -0.11 (0.005) | 195.59 (2.07) |
| 10 | 580.14 (67.69) | -0.0281 (0.003) | -0.0015 (0.004) | 10.58 (0.21) | -0.11 (0.005) | 205.79 (2.12) |

| **Table S8**. Group selection on average body weight | | | | | | |
| --- | --- | --- | --- | --- | --- | --- |
|  | | | | | | |
| Generation | Variability | Average $A_{D}$ | Average $A_{I}$ | Average $A_{GR}$ | Average *b* | Average body weight |
|  | | | | | | |
| base | 376.89 (29.15) | -0.0001 (0.0003) | -0.00002 (0.0003) | 0.01 (0.002) | -0.08 (0.0003) | 100.1 (0.38) |
| 1 | 363.49 (16.49) | -0.0002 (0.001) | -0.0002 (0.001) | 0.95 (0.07) | -0.08 (0.001) | 109.59 (0.67) |
| 2 | 354.95 ( 21.38) | -0.0010 (0.002) | 0.0003 (0.001) | 1.81 (0.09) | -0.08 (0.002) | 118.21 (0.89) |
| 3 | 361.34 (25.35) | -0.0017 (0.002) | 0.0004 (0.002) | 2.67 (0.09) | -0.08 (0.003) | 126.73 (0.98) |
| 4 | 370.77 (29.09) | -0.0029 (0.002) | 0.0006 (0.002) | 3.51 (0.11) | -0.08 (0.003) | 135.19 (1.01) |
| 5 | 375.98 (29.89) | -0.0040 (0.002) | 0.0006 (0.002) | 4.37 (0.13) | -0.08 (0.003) | 143.80 (1.33) |
| 6 | 385.48 (34.45) | -0.0050 (0.003) | 0.0008 (0.003) | 5.23 (0.14) | -0.08 (0.003) | 152.35 (1.42) |
| 7 | 394.274 (39.34) | -0.0062 (0.003) | 0.0007 (0.003) | 6.08 (0.15) | -0.09 (0.004) | 160.89 (1.40) |
| 8 | 404.00 (43.31) | -0.0074 (0.003) | 0.0006 (0.003) | 6.93 (0.16) | -0.09 (0.004) | 169.42 (1.63) |
| 9 | 415.67 (46.07) | -0.0086 (0.003) | 0.0007 (0.003) | 7.79 (0.18) | -0.09 (0.004) | 178.02 (1.78) |
| 10 | 429.29 (50.59) | -0.0097 (0.004) | 0.0006 (0.003) | 8.64 (0.19) | -0.09 (0.005) | 186.46 (1.88) |

| **Table S9**. Group selection on variance of body weight | | | | | | |
| --- | --- | --- | --- | --- | --- | --- |
|  | | | | | | |
| Generation | Variability | Average $A_{D}$ | Average $A_{I}$ | Average $A_{GR}$ | Average *b* | Average body weight |
|  | | | | | | |
| base | 376.89 (29.15) | -0.00006 (0.0003) | -0.00002 (0.0003) | 0.01 (0.002) | -0.08 (0.0003) | 100.1 (0.38) |
| 1 | 321.72 (15.96) | 0.0009 (0.001) | 0.0010 (0.001) | -0.007 (0.07) | -0.08 (0.001) | 99.9 (0.70) |
| 2 | 301.54 (15.27) | 0.0020 (0.001) | 0.0010 (0.001) | 0.0008 (0.08) | -0.08 (0.002) | 100 (0.84) |
| 3 | 291.06 (14.36) | 0.0031 (0.002) | 0.0011 (0.001) | 0.004 (0.09) | -0.08 (0.002) | 100.1 (0.95) |
| 4 | 284.19 (15.29) | 0.004 (0.002) | 0.0011 (0.002) | 0.008 (0.09) | -0.07 (0.002) | 100.1 (0.90) |
| 5 | 277.10 (15.02) | 0.005 (0.002) | 0.0011 (0.002) | 0.007 (0.11) | -0.07 (0.003) | 100 (1.06) |
| 6 | 272.18 (17.24) | 0.006 (0.002) | 0.0011 (0.002) | 0.004 (0.11) | -0.07 (0.002) | 100.1 (1.13) |
| 7 | 268.64 (17.91) | 0.008 (0.002) | 0.0011 (0.002) | 0.006 (0.12) | -0.07 (0.003) | 100 (1.19) |
| 8 | 262.72 (18.48) | 0.009 (0.002) | 0.0011 (0.003) | 0.002 (0.12) | -0.07 (0.003) | 100.1 (1.23) |
| 9 | 255.94 (18.99) | 0.010 (0.002) | 0.001 (0.003) | 0.009 (0.13) | -0.06 (0.003) | 100.1 (1.34) |
| 10 | 250.18 (21.47) | 0.011 (0.003) | 0.0011 (0.003) | 0.008 (0.13) | -0.06 (0.003) | 100.1 (1.36) |
